# A new species of the genus *Australobius* Chamberlin, 1920 (Lithobiomorpha, Lithobiidae) from southwestern China

**DOI:** 10.3897/BDJ.14.e182797

**Published:** 2026-01-22

**Authors:** Yutong Zhang, Huiqin Ma, Sujian Pei, Feng Zhang

**Affiliations:** 1 Hebei Basic Science Center for Biotic Interaction, Hebei University, Baoding, China Hebei Basic Science Center for Biotic Interaction, Hebei University Baoding China; 2 Key Laboratory of Zoological Systematics and Application, College of Life Sciences, Hebei University, Baoding, China Key Laboratory of Zoological Systematics and Application, College of Life Sciences, Hebei University Baoding China; 3 Hebei University, Baoding, China Hebei University Baoding China; 4 State Key Laboratory for Quality Ensurance and Sustainable Use of Dao-di Herbs, Beijing, China State Key Laboratory for Quality Ensurance and Sustainable Use of Dao-di Herbs Beijing China; 5 School of Life Sciences, Hengshui University, Hengshui, China School of Life Sciences, Hengshui University Hengshui China; 6 Hebei Key Laboratory of Wetland Ecology and Conservation, Hengshui, China Hebei Key Laboratory of Wetland Ecology and Conservation Hengshui China

**Keywords:** Chilopoda, new specices, taxonomy, Chongqing

## Abstract

**Background:**

The genus *Australobius* Chamberlin, 1920 is a small genus of subfamily Lithobiinae Newport, 1844 within family Lithobiidae Newport, 1844. The World Catalogue of Centipedes currently records 35 species or subspecies for *Australobius*.

**New information:**

A new species *Australobius septforaminis* sp. nov., is described based on both sexes (four males and two females specimens) from Guanshan, Yintiaoling National Nature Reserve, Wuxi County, Chongqing, China. A detailed morphological description and illustrations of *Australobius septforaminis* sp. nov. are provided.

## Introduction

Chamberlin (1920) established the genus *Australobius* by a single male individual, with the type species *Australobius scabrior* Chamberlin, 1920 from Australia. Attems created the genus *Tamulinus* (Attems 1926). Later, Attems revised the taxonomic status *Australobius* and *Tamulinus* to subgenera of *Lithobius* Leach, 1814 (*Bonato et al. 2016*); Eason (1981) elevated *Australobius* to the rank of genus. The species of *Australobius* is mostly distributed in south-east Asia and eastern Australia, occurring in temperate montane forests to alpine meadows (up to 4850 m a.s.l.) and rainforests to wet sclerophyll forests (from near sea level to 1560 m in Australia) (Zapparoli and Edgecombe 2011). The World Catalogue of Centipedes currently records 35 species or subspecies for *Australobius* (*Bonato et al. 2016*). Altogether, eight species of *Australobius* have been recorded from China (Ma et al. 2008a, Ma et al. 2008b, Ma et al. 2014, Qin et al. 2017). The species and their distribution in China as follows: *A. anamagnus* Ma, Song & Zhu, 2008, from Xizang; *A. apicicornis* Qin, Lin, Zhao, 2014, from Sichuan; *A. cangshanensis* Chao, Lee & Yang, 2020, from Yunan; *A. magnus* (Trozina, 1894), in Xinjiang; *A. nodulus* Ma, Song & Zhu, 2008, from Xizang; *A. polyspinipes* Ma, Liu & Lu, 2018, from Hebei; *A. tetrophthalmus* (Loksa, 1959), from Guangxi, and *A. tracheoperspicuus* Li, Pei & Guo, 2018, from Guizhou (Bonato et al. 2016).

*Australobius* characterised by the following combination of characters: antennae mostly with 21–22 segments, while some species with more than 24 segments; ocelli large, conspicuous and few in number (mostly 4–7, with some species having more than 8); Coxosternite usually with 5+5 teeth and without prominent setae or spines on the surface; the last four pairs of legs bear only a single row of coxal pores, and the coxae lack spines on their lateral and ventral surfaces; legs 15 weakly armed with a small dorsal spine; the ventral plectrotaxy 01320, and the dorsal plectrotaxy 12200; male legs 15 simply enlarged without any prominent projections.

Myriapoda fauna of China still poorly known and this especially the case with centipedes of the order Lithobiomorpha. In total, 113 species/subspecies of lithobiomorphs to date known from China (Ma et al. 2014, Pei et al. 2015a, Pei et al. 2018, Pei et al. 2015b, PeiI et al. 2016, Chao et al. 2018, Qiao et al. 2018). Southwestern China represents one of the world’s biodiversity hotspots (Bonato et al. 2016). However, research on myriapoda diversity in this region remains remarkably scarce. Recent surveys on *Australobius* in China suggest that our understanding of its species diversity remains far from complete, with many species still awaiting description (Bonato et al. 2016). The present study represents a part of our investigation into *Australobius* diversity in China, deals with the description and illustration of a new species of the genus from the Yintiaoling National Nature Reserve in Wuxi County, Chongqing Municipality, southwestern China. A map of collecting localities and an identification key to Chinese *Australobius* species based on adult specimens are presented.

## Materials and methods

All specimens collected from leaf litter or beneath stones using forceps and preserved in 75% or 95% ethanol, respectively, for the purpose of meeting morphological and molecular biological research requirements. All specimens examined using a Leica M205A. Images taken using a Leica M205A stereomicroscope, equipped with a DFC550 CCD camera, or an Olympus BX53 microscope, equipped with a Kuy Nice CCD camera and imported into Helicon Focus Ver. 7.0 for stacking. Plates and photographs were edited and retouched using Adobe Photoshop 2022. The distribution map made using QGIS 3.34.10. The anatomical terminology follows the proposed terminology used in this paper (Bonato et al. 2010). The color description based on specimens preserved in 75% ethanol. Body length measured from the anterior margin of the cephalic plate to the posterior end of the postpedal tergite. Specimens deposited in Institute of Myriapodology, School of Life Sciences, Hengshui University, Hengshui, P. R. China (HUSLSIM).

Abbreviations as follows: A: anterior; C: coxa; F: femur; m: median; p: posterior; P: prefemur; S, SS: sternite, sternites; T, TT: tergite, tergites; Tr: trochanter; Ti: tibia.

## Taxon treatments

### Australobius septforaminis

Zhang, Ma, Pei & Zhang sp. nov.

E012A72B-5779-50E7-BBDC-208A36A1A6D5

7EC29B22-C5B8-44FF-A39D-797CF903BD19

#### Materials

- **Type status:** Holotype. **Occurrence:** recordedBy: Ma Hui-qin, et al.; individualCount: 1; sex: male; lifeStage: adult; occurrenceID: 7F9B9D77-57BF-5917-8E34-B9D91103B75D; **Taxon:** scientificName: *Australobius septforaminis*; kingdom: Animalia; phylum: Arthropoda; class: Chilopoda; order: Lithobiomorpha; family: Lithobiidae; genus: Australobius; taxonRank: species; taxonomicStatus: species; **Location:** continent: Asia; country: China; stateProvince: Chongqing Municipality; county: Wuxi; locality: Yintiaoling National Nature Reserve; verbatimElevation: 1844 m; verbatimCoordinates: 31°28′25.45′′N 109°47′6.97′′E; decimalLatitude: 31.473736; decimalLongitude: 109.785269; georeferenceProtocol: label; **Identification:** identifiedBy: ZhangYu-tong; dateIdentified: 2025; **Record Level:** collectionCode: myriapoda; basisOfRecord: Preserved Specimen
- **Type status:** Paratype. **Occurrence:** recordedBy: Ma Hui-qin, et al.; individualCount: 2; sex: female; lifeStage: adult; occurrenceID: B81F157F-85E8-5FF9-8D01-D95F347A0415; **Taxon:** scientificName: *Australobius septforaminis*; kingdom: Animalia; phylum: Arthropoda; class: Chilopoda; order: Lithobiomorpha; family: Lithobiidae; genus: Australobius; taxonRank: species; taxonomicStatus: species; **Location:** continent: Asia; country: China; stateProvince: Chongqing Municipality; county: Wuxi; locality: Yintiaoling National Nature Reserve; verbatimElevation: 1844 m; verbatimCoordinates: 31°28′25.45′′N 109°47′6.97′′E; decimalLatitude: 31.473736; decimalLongitude: 109.785269; georeferenceProtocol: label; **Identification:** identifiedBy: ZhangYu-tong; dateIdentified: 2025; **Record Level:** collectionCode: myriapoda; basisOfRecord: Preserved Specimen
- **Type status:** Other material. **Occurrence:** recordedBy: Ma Hui-qin, et al.; individualCount: 2; sex: male; lifeStage: adult; occurrenceID: B177F05E-BD8D-5759-AEE1-8E5BAED009DE; **Taxon:** scientificName: *Australobius septforaminis*; kingdom: Animalia; phylum: Arthropoda; class: Chilopoda; order: Lithobiomorpha; family: Lithobiidae; genus: Australobius; taxonRank: species; taxonomicStatus: species; **Location:** continent: Asia; country: China; stateProvince: Chongqing Municipality; county: Wuxi; locality: Yintiaoling National Nature Reserve; verbatimElevation: 1844 m; verbatimCoordinates: 31°28′25.45′′N, 109°47′6.97′′E; decimalLatitude: 31.473736; decimalLongitude: 109.785269; georeferenceProtocol: label; **Identification:** identifiedBy: ZhangYu-tong; dateIdentified: 2025; **Record Level:** collectionCode: myriapoda; basisOfRecord: Preserved Specimen

#### Description

**Figure 1.**
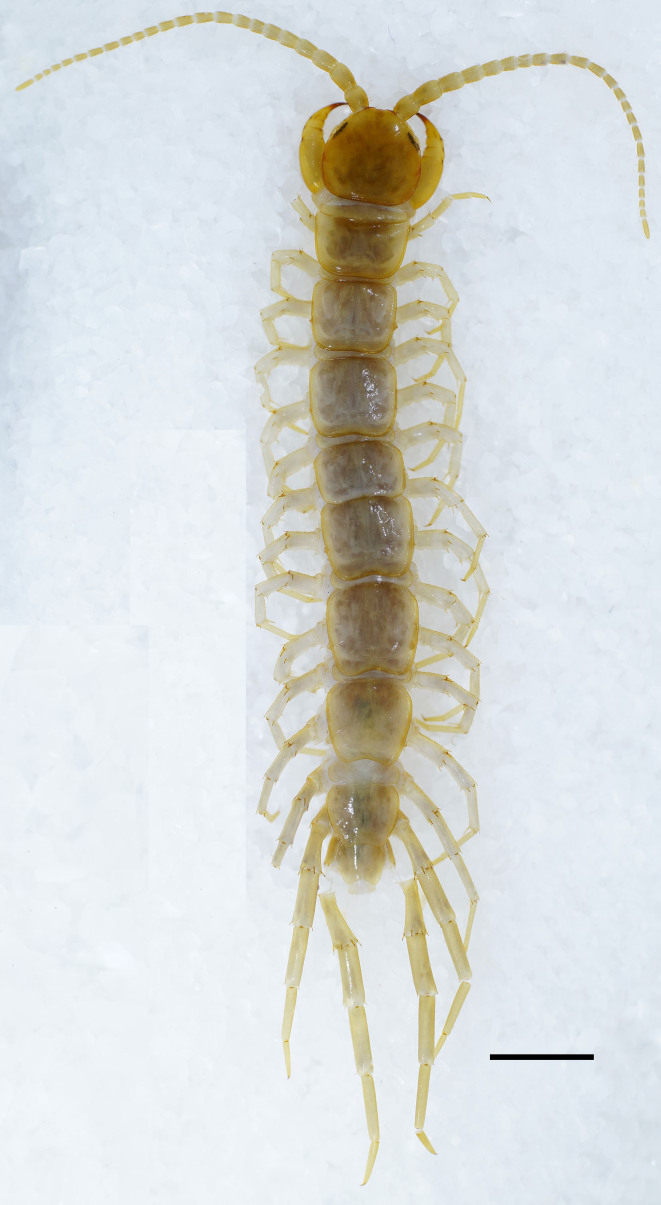
*Australobius septforaminis* sp. nov., male holotype, habitus, dorsal view. Scale bars = 2 mm.

**Body**: Measurements (male holotype, Fig. 1). Holotype body 26.60 mm long, cephalic plate 2.41 mm long, 2.62 mm wide. Body length 15.90–26.60 mm. Head length 1.72–2.41 mm, width 1.79–2.62 mm.

**Color**: The basal segments of the antennae yellowish–brown. Tergites yellowish–brown, with the head shield, TT1 and 14 and the last tergite yellowish–brown, T 1 particularly dark yellowish–brown, and the cephalic plate red. Transverse groove of the cephalic plate slightly posterior to the anterior end of the cephalic plate and yellowish in color, becoming more yellowish–brown towards posterior. Pleural region light gray to dark gray. The ventral side light yellowish–brown. Forcipular tarsungulum dark brown, while the rest of the Forcipular tarsungulum, coxosternite, S 15 and ventral plate of the pregenital segment yellowish–brown. Legs 1–13 light yellowish–brown, with the tarsal segments of each leg yellowish–brown.

**Antennae**: Mostly 23 articles (few 24), holotype 23+23 articles. The first three basal articles of the antennae significantly longer than wide. The length of the subsequent articles gradually decreases, with the final article significantly longer than wide, 2.3-3.2 times longer than wide (Fig. 1); abundant setae on the antennal surface, fewer on the basal articles, gradual increasing in density to approximately the fourth article, then more or less constant.

**Figure 2.**
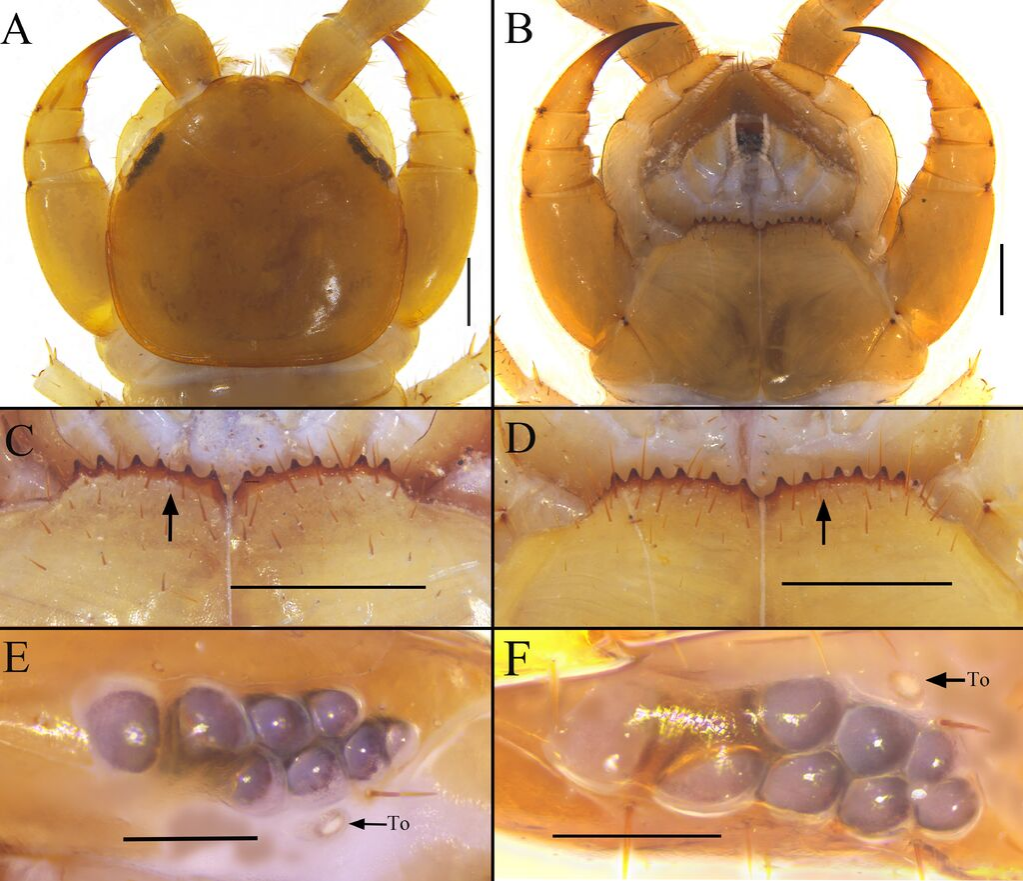
*Australobius septforaminis* sp. nov., Male holotype (A–C, E), female (D, F). **A** cephalic plate, dorsal view; **B–D** forcipular coxosternite, ventral view; **E** ocelli and Tömösváry’s organ (To); **F** ocelli and Tömösváry’s organ (To). Scale bars = 0.5 mm (A, B, C, D, E, F).

**Cephalic plate**: Anterior margin of the cephalic plate slightly concave central, with a distinct transverse groove, slightly broader than length. Tiny setae scattered sparsely over the whole surface, the margins of the cephalic plate with very sparsely distributed, nearly uniformly long setae, and the whole surface of cephalic plate is covered with a fine hexagonal mesh. The lateral margin of the cephalic capsule only begins to with a ridge from the side to the midpoint between the ocelli and the posterior margin, with a slightly concave posterior margin and a complete ridge (Fig. 2A).

**Ocelli**: 8–9, usually 8, arranged in two irregular rows, 5+3 or 4+4, in one female specimen with nine ocelli on the right side, arranged in a 6+3 pattern, while the left side with eight ocelli, arranged in a 5+3 pattern; oval to rounded ocelli on each side, the posterior two ocelli larger. The ocelli near the Tömösváry’s organ smaller, while the other ocelli nearly equal in size. Mostly translucent, with black pigmentation at the base, and hemispherical in shape, with a slightly darker color in the eye area.

**Tömösváry’s organ**: Close to the ocelli, situated at the anterolateral margin of the cephalic plate, on the ventral side of the anterior half of the eye area. The Tömösváry’s organ small, nearly round, the surrounding sclerotised area wide, and slightly smaller than the adjoining ocelli (Fig. 2E, F).

**Coxosternite**: Subtrapezoidal. Lateral margins moderately shorter than the medial margins. Anterior margin wide and convex forward, presenting a typical bow shape. Median diastema deep, a slightly deeper V shape (Fig. 2C). Whole surface of coxosternite covered with fine hexagonal mesh. Anterior margin bearing 6–7 blunt triangular teeth, usually 6+6, rarely 6+7. Outermost tooth with distinct shoulder on outer side, followed by gradual retreat. Porodonts slightly thicker, almost transparent, located between second and third inner teeth, without basal bulge (Fig. 2C–E, F). Long scattered setae on ventral side of coxosternite, longer setae near dental margin (Fig. 2B–D).

**Tergites**: Smooth, slightly convex; short to long tiny setae emerging from pores scattered sparsely over the entire surface, near the margin with few long setae; T1 wider than the cephalic plate and slightly wider than T3. T1 narrower postero-laterally than antero-laterally, generally inverted trapezoidal. The lateral marginal ridges of all tergites continuous, posterior marginal ridge of TT1, 3 and 5 continuous, posterior marginal ridge of TT 8, 10, 12 and 14 discontinuous. The posterior margin of TT1 and 3 slightly concave, posterior margin of T5 concave, posterior margin of TT7, 8, 10, 12 and 14 concave (Fig. 1). The posterior angles of tergites 9, 11, and 13 with very slightly triangular projections. The lateral margins of the tergites with sparse short setae at the anterior angles, and 1–2 long setae, and 1–2 longer setae at the posterior angles (Fig. 1).

**Sternites**: Posterior side of sternites narrower than anterior, generally inverted trapezoidal, smooth; setae on the surface and lateral margin, very few short setae scattered sparsely amongst them, with more setae at both the anterior and the posterior corners, and 4–6 longer setae on both middle of anterolateral margins, 1–2 longer setae on middle of both anterolateral margins, there a pair of long setae that nearly symmetrically distributed; S14 has more dense setae, especially in the S15, from long to longer setae on the posterior margin.

**Legs**: Relatively robust, tarsi ill-defined on legs 1–13, well-defined on legs 14–15. All legs with moderately long curved claws. Legs 1–13 each carry an anterior accessory spur and a posterior accessory spur: the anterior spur moderately longer and slender, forming a moderately small angle with the claw; the posterior spur slightly longer and more robust, forming a comparatively large angle with the claw. Legs 14 and 15 lack accessory spurs. Short to long setae very sparsely scattered over the surface of the coxa, trochanter, prefemur, femur and tibia of legs 1–13. Tarsal surfaces bear more setae, especially on the ventral side; setae on the dorsal and ventral surfaces slightly longer than those on the anterior and posterior edges. Some obvious thicker setae arranged in one row on the anterior surfaces and in two rows on the ventral surfaces of the tarsi of legs 1–13, except legs 14 and 15, with a shallow groove between the rows of setae. Legs 14 and 15 have few setae that are significantly uniform and homogeneous, and they have dense glandular pores on the inside from femur to tibia. Legs 14 and 15 longer and thicker than the anterior legs in both female and male; male legs 15 moderately thicker and stronger than those of the female. Tarsal articles 2 6.2–6.6 times longer than wide, Tarsal articles 2 67.2%–79.4% length of Tarsal articles 1 on legs 15 in female; Tarsal articles 2 6.3–8.5 times longer than wide, Tarsal articles 2 75.0%–77.6% length of Tarsal articles 1 on legs 15 in male. Leg plectrotaxy given in Table 1 and Table 2.

**Figure 3.**
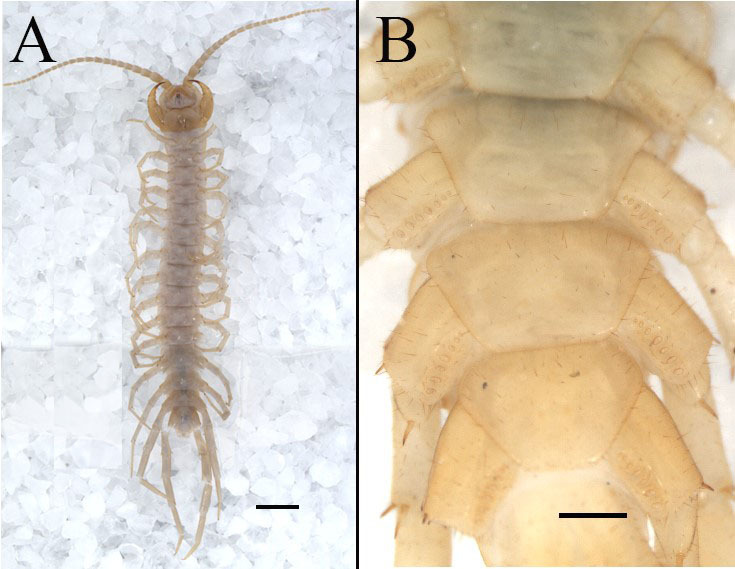
*Australobius septforaminis* sp. nov., male holotype (A), other female(B). **A** habitus, ventral view; **B** SS 14-15 Scale bars = 2 mm (A), scale bars = 1 mm (B).

**Coxal pores**: 6–7 in number, elliptical to subcircular, markedly unequal in size and arranged in a single longitudinal row. Female pattern predominantly 7777, less frequently 6777; male pattern 6785 or 6676 (Fig. 3). The coxal pore field distinctly sunken, forming a shallow groove with elevated margins that bear sparsely distributed setae of varying lengths.

**Figure 4.**
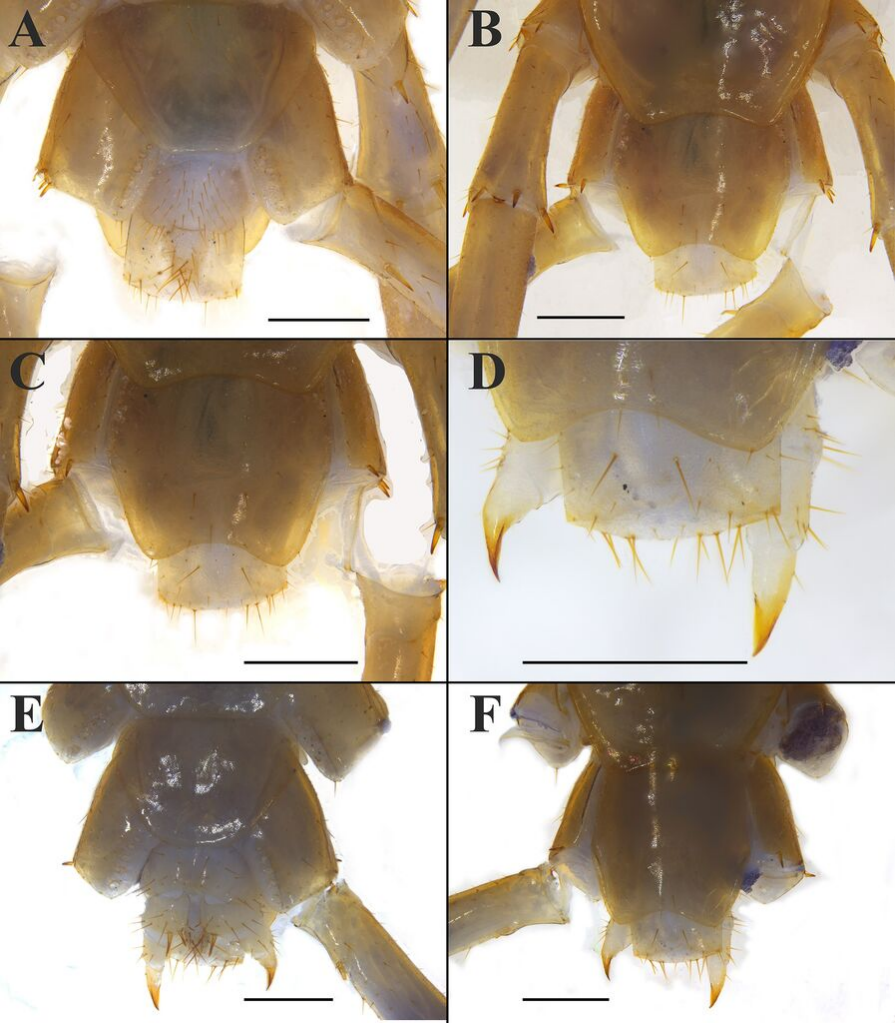
*Australobius septforaminis* sp. nov., Male holotype (A, C) and female (B, D). **A** posterior segments and gonopods, ventral view; **B** posterior segments and gonopods, ventral view; **C** posterior segments and gonopods, magnified dorsal view; **D** posterior segments and gonopods, magnified dorsal view. Scale bars = 0.5 mm (A, B, C, D).

**Female**: S15 anterior margin broader than posterior, generally nearly semi-circular, posterior angles rounded, postero-medially straight. Moderately long to short setae relatively densely scattered on S15 surface. Surface of the lateral sternal margin of pregenital segment well chitinised, posterior margin of pregenital sternite deeply concave between condyles of gonopods, except for a small, median square-shaped bulge. Relatively long setae very sparsely scattered over ventral surface of the pregenital segment, slightly more setae on posterior part, especially along the posterior edge. Gonopods: first article fairly broad, bearing 20–28 moderately long setae, arranged in approximately four irregular longitudinal rows, the setae gradually increasing in number from anterior to posterior, with 4+4 or 3+3 small blunt coniform gonopodal spurs, inner spurs slightly smaller and more anterior than the outer; second article with 10–12 long setae in the ventral, arranged in approximately four irregular rows. Third article with 4–6 longer setae on the ventral surface, arranged in two rows (Fig. 4A-C).

**Male**: S15 posterior margin narrower than anterior, postero-medially slightly convex, generally an inverted trapezoid, sparsely covered with long to short setae, the setae on the edges being longer; the pregenital sternite segment evidently smaller than the female, usually well sclerotized, ventral side obviously convex; posterior margin shallow concave between the gonopods, without medial bulge. Short to long setae equally scattered on the ventral surface of the pregenital segment. Gonopods short and wide, hemispherical, with a single long seta, apically slightly sclerotised (Fig. 4A, C).

#### Diagnosis

In accordance with the grouping of species proposed in the genus *Australobius*, the new species differs from other congeners in combination of characters, by having the antennae composed of 23–24 articles, commonly 23+23 articles, ocelli 8–9, usually 8 on each side, arranged in two rows, with the last two ocelli being the largest, Tömösváry’s organ slightly smaller than the adjacent ocelli; commonly 6+7 coxosternal teeth, porodonts lying between the second and third inner teeth; tergites without posterior triangular projections; coxal pore formula 6–7. Female gonopods with 3+3 or 4+4 moderately small coniform spurs.

#### Etymology

The specific epithet, meaning the last four pairs of legs with seven coxal pore plates respectively.

#### Notes

To facilitate the identification of Chinese *Australobius* species, a map showing the collecting sites of the seven Chinese species is also given (Fig. 6). Because no material of *A. magnus* and *A. tetrophthalmus* was examined and their original descriptions lack precise coordinates, these two species are not plotted on the map.

#### Habitat

**Figure 5.**
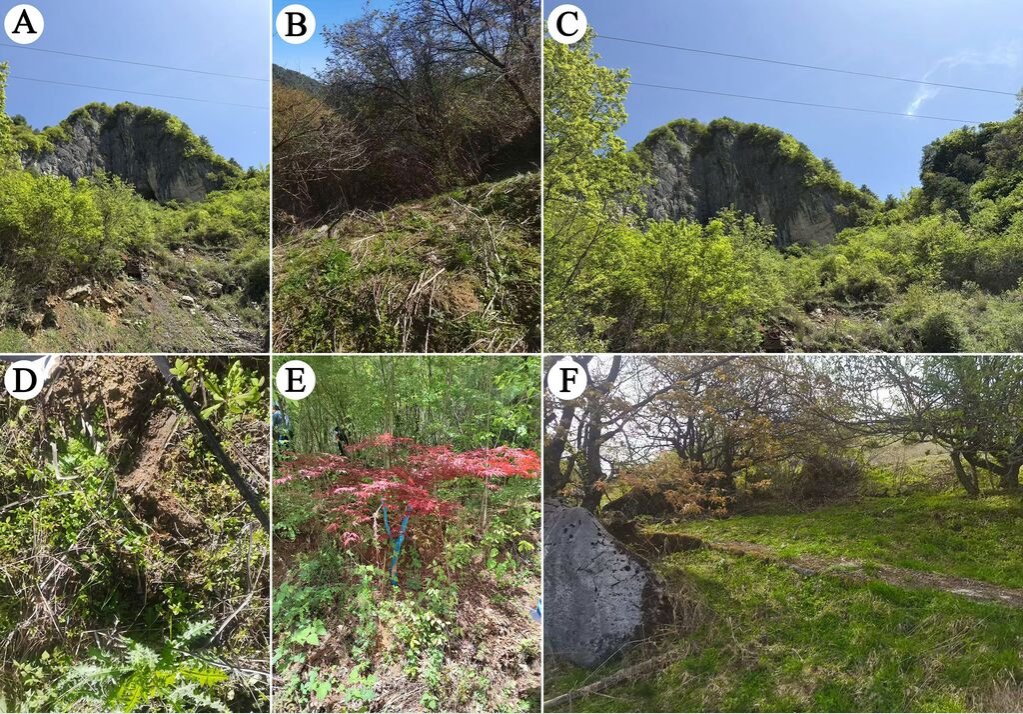
The habitat photos of *Australobius septforaminis* sp. nov.

**Figure 6.**
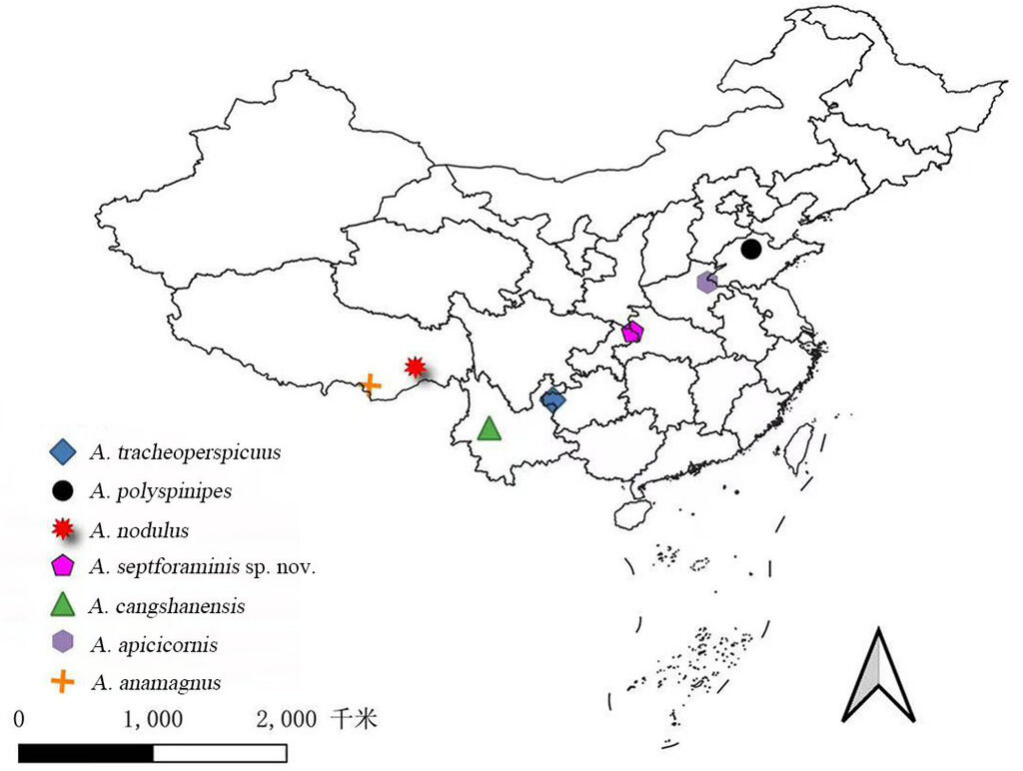
The collecting localities map of Chinese records species of *Australobius* Chamberlin, 1920, expect *Australobius magnus* (Trozina, 1894) and *A. tetrophthalmus* (Loksa, 1959).

The specimens were collected under the bark of trees along the mountain road (Fig. 5A-F).

## Identification Keys

### Key to the Chinese species of *Australobius* Chamberlin, 1920

**Table:** 

1	No ocelli on cephalic plate	*A. tracheoperspicuus* (Li, Pei & Guo, 2018)
–	At least four ocelli on each side of cephalic plate	2
2	Four ocelli on each side of cephalic plate, Tömösváry’s organ larger than adjacent ocelli	*A. tetrophthalmus* (Loksa, 1959)
–	More than seven ocelli on each side of cephalic plate, Tömösváry’s organ smaller than adjacent ocelli	3
3	No porodonts	*A. apicicornis* (Qin, Lin & Zhao, 2014)
–	Porodonts present	4
4	Large posterior tergites wrinkled; a bulge present on terminal of tibiae of 15^th^ legs in male	*A. magnus* (Trozina, 1894)
–	Large posterior tergites smooth; no bulge on the terminal of male 15^th^ tibiae	5
5	Antenna with 31 articles	*A. nodulus* (Ma, Song & Zhu, 2008)
–	Antenna with less than 30 articles	6
6	Antenna with 29 articles	*A. polyspinipes* (Ma, Liu & Lu, 2018)
–	Antenna with less than 27 articles	7
7	Antenna with 26 articles	*A. anamagnus* (Ma, Song & Zhu, 2008)
–	Antenna with 23–24 articles	8
8	the porodonts lying between the fourth and fifth or fifth and sixth outer tooth	*A. cangshanensis* (Chao, Lee & Yang, 2020)
–	the porodonts lying between the second and third inner teeth	*A. septforaminis* sp. nov.

## Discussion

Morphologically, the new species is close to *A. apicicornis* in having ocelli arranged in two regular rows (eight on each side in *A. apicicornis*) and a Tömösváry’s organ smaller than the adjoining ocelli. It can be readily distinguished from *A. apicicornis* by the following characters: porodonts present between the second and third inner teeth versus completely absent in *A. apicicornis*; antennae 23+23 articles versus 24+24 in *A. apicicornis*; All tergites without wrinkles versus all tergites wrinkled in *A. apicicornis*.

In general appearance the new species is close to *A. magnus* in having a Tömösváry’s organ smaller than adjacent ocelli and smooth posterior tergites in both sexes. Distinguishing characters are: porodonts between the second and third inner teeth versus between the first and second inner teeth in *A. magnus*; anterior margin of the coxosternite with 6+7 teeth versus 2+2–5+5 in *A. magnus*; antennae 23+23 articles versus 25–30 in *A. magnus*; Large posterior tergites smooth, no bulge on the terminal of male 15^th^ tibiae versus large posterior tergites wrinkled, a bulge present on terminal of tibiae of 15^th^ legs in male in *A. magnus*.

The new species closely resembles *A. nodulus* in possessing smooth posterior tergites and a Tömösváry’s organ smaller than adjoining ocelli. It differs as follows: porodonts between the second and third inner teeth versus between the third and fourth inner teeth in *A. nodulus*; ocelli 8 in two regular rows versus 9–11 in *A. nodulus*; antennae 23+23 articles versus 31–33 in *A. nodulus*.

Morphologically, the new species is similar to *A. polyspinipes* in having smooth posterior tergites, a Tömösváry’s organ smaller than adjacent ocelli, and a comparatively large posteriormost ocellus. It can be distinguished by: porodonts between the second and third inner teeth versus between the fourth and fifth inner teeth (eight coxosternal teeth) and between the third and fourth inner teeth (seven coxosternal teeth) in *A. polyspinipes*; ocelli 8 versus 1+11 in *A. polyspinipes*; antennae 23+23 articles versus 29+29 in *A. polyspinipes*.

The new species and *A. anamagnus* share smooth posterior tergites and the absence of an apical bulge on the male fifteenth tibia. Diagnostic differences are: porodonts between the second and third inner teeth versus between the first and second inner teeth (three coxosternal teeth) and between the second and third inter teeth (four coxosternal teeth) in *A. anamagnus*; ocelli eight in two regular rows versus ten with only the posteriormost ocellus enlarged in *A. anamagnus*; anterior margin of the coxosternite 6+7 teeth versus 3+3 - 3+4 in *A. anamagnus*; antennae 23+23 articles versus 26 (rarely 25+26) in *A. anamagnus*.

Compared with *A. cangshanensis*, the new species shares 6–7 pairs of small, blunt prosternal teeth, ocelli in two regular rows with the posterior ocellus slightly larger, a Tömösváry’s organ smaller than adjoining ocelli, and smooth posterior tergites. It is readily distinguished by: anterior margin of the coxosternite with 6+7 teeth versus 7–10 in *A. cangshanensis*; female gonopodial spurs arranged 2+2 versus 3+4 in *A. cangshanensis*; porodonts between the second and third inner teeth versus between the fourth and fifth or fifth and sixth outer teeth in *A. cangshanensis*.

The new species is separated from *A. tracheoperspicuus* by: porodonts present between the second and third inner teeth versus completely absent in *A. tracheoperspicuus*; ocelli eight in two regular rows versus absent in *A. tracheoperspicuus*; anterior margin of the coxosternite 6+7 teeth versus 5+5 in *A. tracheoperspicuus*; antennae 23+23 articles versus 26+26 in *A. tracheoperspicuus*.

Compared with *A. tetrophthalmus*, the new species is readily distinguished by: antennae 23+23 versus 29 in *A. tetrophthalmus*; ocelli arranged in two regular rows, 8–9 (usually 8), with a Tömösváry’s organ smaller than adjoining ocelli versus four ocelli (two large and two small) and a Tömösváry’s organ larger than adjoining ocelli in *A. tetrophthalmus*; anterior margin of the coxosternite with 6+7 teeth versus 5+5 in *A. tetrophthalmus*; coxal pores 7 versus 3 in *A. tetrophthalmus*.

## Supplementary Material

XML Treatment for Australobius septforaminis

## Figures and Tables

**Table 1.** Leg plectrotaxy of *Australobius septforaminis* sp. nov., males.

legs	dorsal					ventral				
	C	Tr	P	F	Ti	C	Tr	P	F	Ti
1			amp	amp	amp			amp	ap	ap
2–5			amp	amp	amp			amp	amp	ap
6			amp	amp	amp			amp	amp	amp
7–8			amp	amp	amp			amp	amp	ap
9			amp	amp	amp			amp	amp	amp
10			mp	amp	amp			amp	amp	amp
11–12			mp	amp	amp			amp	amp	amp
13			mp	amp	a	a		amp	ap	amp
14		m	amp	amp	ap	a		amp	amp	p
15	a	m	amp	a	a	ap		amp	ap	a

**Table 2.** Leg plectrotaxy of *Australobius septforaminis* sp. nov., females.

Legs	dorsal					ventral				
	C	Tr	P	F	Ti	C	Tr	P	F	Ti
1			amp	ap	amp			amp	am	amp
2			amp	amp	amp			mp	amp	am
3			amp	amp	amp			amp	amp	ap
4–5			amp	amp	amp			amp	ap	ap
6			amp	amp	amp			amp	ap	p
7			amp	amp	amp			amp	amp	am
8–9			amp	amp	amp			amp	amp	amp
10			amp	amp	amp			ap	ap	amp
11			amp	amp	p			amp	amp	a
12			amp	amp	amp			amp	amp	amp
13		ap	amp	amp	amp			amp	ap	ap
14		m	amp	amp	amp	a		amp	ap	ap
15		m	amp	amp	a			amp	am	p
